# Steroid-refractory dermatologic and pulmonary toxicity in a patient on rituximab treated with pembrolizumab for progressive urothelial carcinoma: a case report

**DOI:** 10.1186/s13256-021-02670-3

**Published:** 2021-03-19

**Authors:** Jacobi Hines, Ellen Daily, Anh Khoa Pham, Christopher R. Shea, Urooba Nadeem, Aliya N. Husain, Walter M. Stadler, Pankti Reid

**Affiliations:** 1 Internal Medicine Residency Program, Department of Medicine, UChicago Medicine, 5841 S Maryland Ave, Ste MC 7082, Chicago, IL 60637-1465 USA; 2 Department of Pathology, UChicago Medicine, Chicago, IL USA; 3 Section of Dermatology, Department of Medicine, UChicago Medicine, Chicago, IL USA; 4 Section of Hematology/Oncology, Department of Medicine, UChicago Medicine, Chicago, IL USA; 5 Section of Rheumatology, Department of Medicine, UChicago Medicine, Chicago, IL USA

**Keywords:** Immunotherapy, Bullous skin diseases, Medical oncology, Case report, Immune-related adverse effects, Immune checkpoint inhibitors

## Abstract

**Background:**

Increasingly widespread use of programmed cell death protein 1 (PD-1) immune checkpoint inhibitors (ICIs) for treatment of a variety of progressive malignancies continues to reveal a range of immune-related adverse events (irAEs), necessitating immunosuppressive therapy for management. While a single course of systemic corticosteroids may be sufficient for many irAEs, no clear standard-of-care guidelines exist for steroid-refractory cases. We present an unusual case of a patient who developed several steroid-refractory novel irAEs on pembrolizumab despite ongoing B cell-directed immunosuppressive therapy with rituximab, who ultimately noted resolution of symptoms with tacrolimus, a T-cell-directed immunosuppressant.

**Case presentation:**

This 72-year-old Caucasian man with Waldenstrom’s macroglobulinemia and myelin-associated glycoprotein (MAG) immunoglobulin M (IgM) antibody-associated neuropathy was being treated with maintenance rituximab and intravenous immunoglobulin when he was started on pembrolizumab (2.26 mg/kg) for metastatic urothelial cancer 31 months after surgery and adjuvant chemotherapy. After his third dose of pembrolizumab, he developed a painful blistering papular rash of the distal extremities. He received two more doses of pembrolizumab before he also developed diarrhea, and it was held; he was initiated on 1 mg/kg prednisone for presumed ICI-induced dermatitis and colitis. Skin biopsy 10 weeks after cessation of pembrolizumab and taper of steroids to 20  mg daily revealed a unique bullous erythema multiforme. He was then admitted with dyspnea and imaging concerning for necrotizing pneumonia, but did not respond to antibiotic therapy. Bronchoscopy and biopsy revealed acute fibrinous organizing pneumonia. His symptoms failed to fully respond to multiple courses of high-dose systemic corticosteroids and a trial of azathioprine, but pneumonia, diarrhea, and skin rash all improved markedly with tacrolimus. The patient has since completed his therapy for tacrolimus, continues off of ICI, and has not experienced a recurrence of any irAEs, though has more recently experienced progression of his cancer.

**Conclusion:**

Despite immunosuppression with rituximab and intravenous immunoglobulin, two immunomodulators targeting B cells, ICI cessation, and systemic corticosteroid therapy, our patient developed two high-grade unusual irAEs, bullous erythema multiforme and acute fibrinous organizing pneumonia. Our patient’s improvement with tacrolimus can offer critical insight into the pathophysiology of steroid-refractory irAEs.

## Background

Programmed cell death protein 1 (PD-1) is an immune checkpoint protein that prevents autoimmunity and quiets the immune response. It is expressed on T cells (CD4^+^ and CD8^+^), B cells, and natural killer (NK) cells. Its ligand, PD-L1, is expressed on a variety of cells including cancer cells, epithelial cells, and endothelial cells. PD-L1 expression is upregulated by certain cytokines and oncogenes which can cause suppression of the immune response against cancer cells [1]. PD-1 monoclonal antibodies such as pembrolizumab and nivolumab inhibit this PD-1/PD-L1 interaction and have thus been incorporated into many therapeutic regimens for a variety of advanced malignancies, including melanoma, lung cancer, hepatocellular carcinoma, renal cell carcinoma, and urothelial carcinoma.

Because of the broad expression of PD-L1 on a variety of non-pathologic human cell types, the PD-1 immune checkpoint inhibitors (ICIs) can result in a range of immune-related adverse effects (irAEs), such as widespread fatigue and devastating myalgias, but also toxicities more akin to autoimmune diseases such as colitis and pneumonitis [2]. The incidence of these irAEs can be as high as 60% [3]. Generally, most of these adverse events can resolve quickly upon cessation of the ICI and initiation of systemic corticosteroids, but certain toxicities can be refractory to even high doses of corticosteroids and can even occur after cessation of cancer immunotherapy with a complex multi-organ presentation [4–7]. The treatment options for these steroid-refractory irAEs are not well known [8, 9]. Tumor necrosis factor alpha inhibitors and azathioprine (inhibitor of purine synthesis) have previously been used [10, 11], and there are limited cases in the literature showing improvement with tacrolimus, a calcineurin inhibitor leading to suppression of T-cell proliferation [12].

We present here a unique case of a patient with prior suppression of the B-cell immune pathway with rituximab, before initiation of an ICI (pembrolizumab). His complex case reveals a unique dermatologic irAE, bullous erythema multiforme, amidst a constellation of other multisystem irAEs including acute fibrinous organizing pneumonia (AFOP), which has not been previously reported in association with pembrolizumab.

## Case presentation

A 72-year-old Caucasian man with a history of Waldenstrom’s macroglobulinemia, immunoglobulin M (IgM) paraproteinemia with high titer of myelin-associated glycoprotein (MAG) IgM antibody, and associated neuropathy was being effectively treated with maintenance rituximab and intravenous immunoglobulin (IVIG) when he was started on pembrolizumab for progression of metastatic urothelial carcinoma 31 months after surgery and adjuvant chemotherapy (gemcitabine, carboplatin, paclitaxel). Of note, he also had a past medical history of hypertension, hyperlipidemia, atrial flutter, and diverticulosis. His family history included melanoma (mother and father), non-Hodgkin lymphoma in his mother, breast cancer in his mother, and adrenal gland cancer in his sister. He was a former smoker for 30 pack-years and had quit 18 years prior to presentation. He also had prior heavy alcohol use, but had been sober for 15 years prior to presentation. At the time of this treatment, he was smoking marijuana several times per month.

**Fig. 1:**
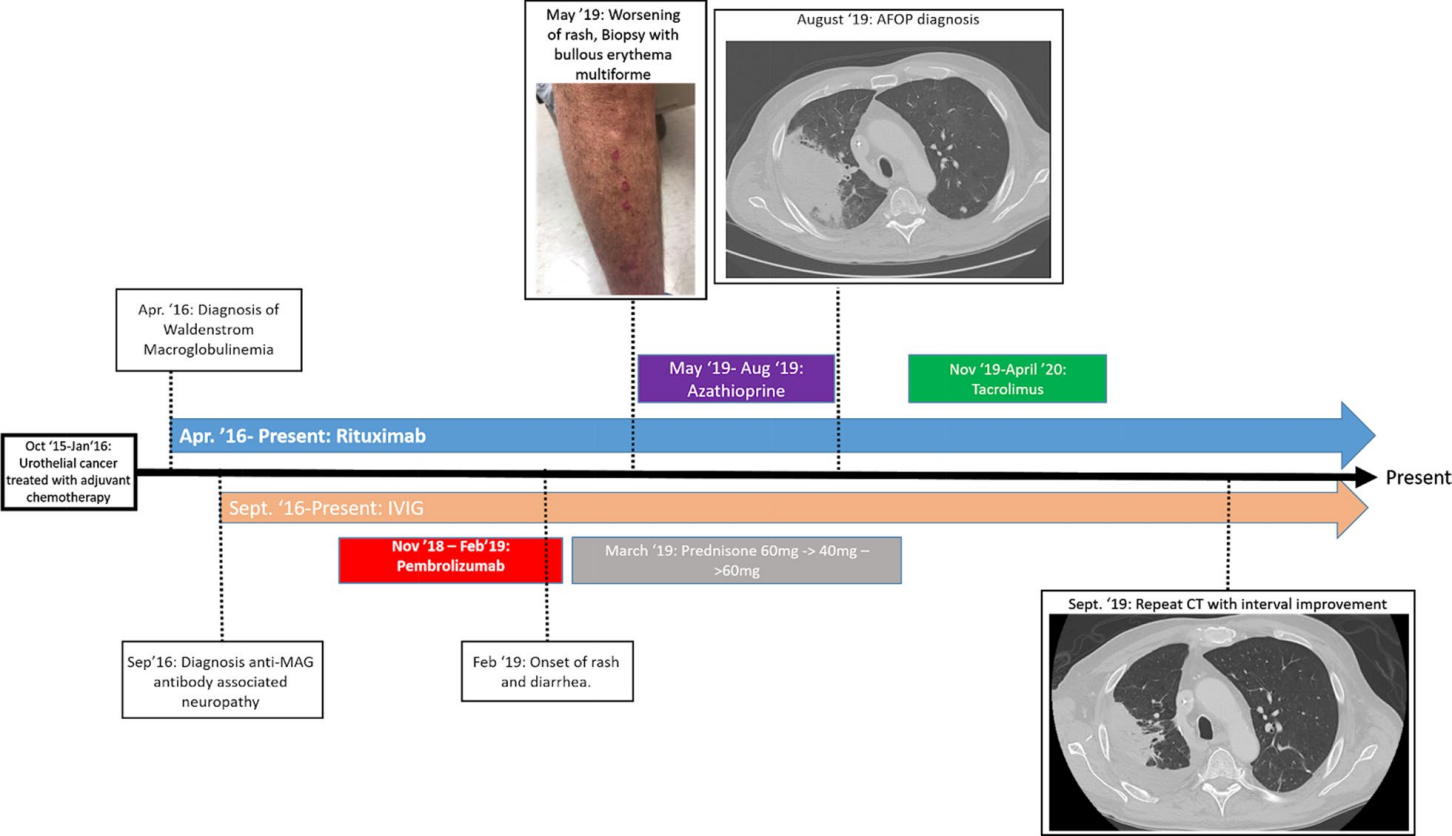
Timeline of clinical course. Visual representation of patient’s diagnosis and initiation of therapy, with insight into onset of dermatologic and pulmonary irAEs (representative images at time of diagnosis) and attempted therapies. Follow-up chest computed tomography findings are shown. Follow-up exam of skin showed nearly complete resolution of initial findings, but was not documented in photograph

He tolerated five total cycles/doses of pembrolizumab (200 mg/dose), initially only experiencing transient neutropenia (absolute neutrophil count 120) and thrombocytopenia (platelet count 121,000). However, after his third dose, he noted a new rash on his chest and two lip lesions. The rash consisted of red/purple blistering papular rash with associated pain, most predominant in the distal extremities (Fig. 1). Over the next month, he had persistent skin changes and new onset of significant diarrhea. Pembrolizumab was discontinued and his symptoms improved after 2 weeks of prednisone 60mg daily.

**Fig. 2:**
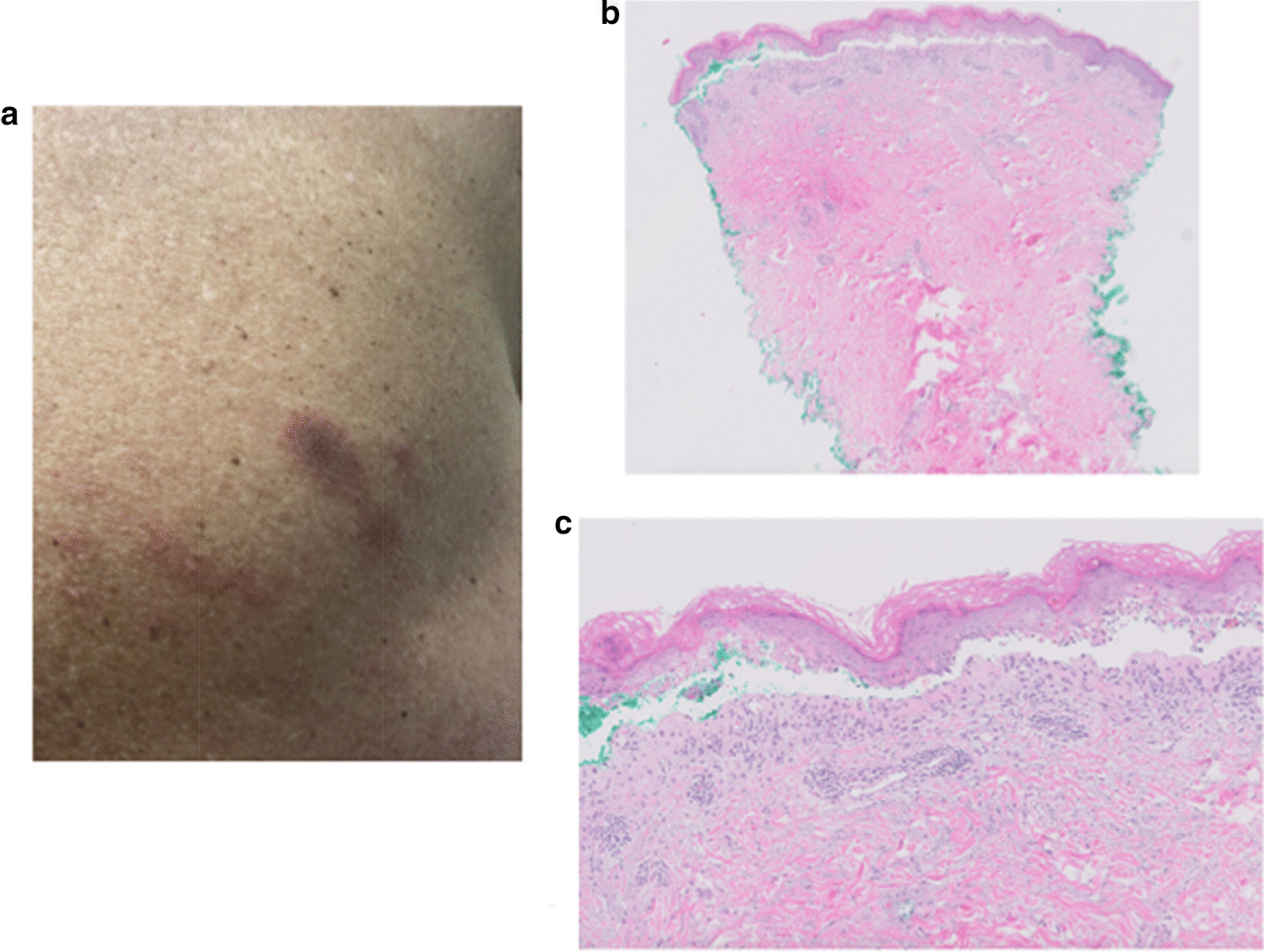
**a** Site of punch biopsy on patient’s upper back. **b** At scanning magnification, there is a basket-weave stratum corneum with a subepidermal split and mild superficial perivascular inflammation (hematoxylin and eosin [H&E], original magnification ×10). **c** On higher magnification, there is an interface dermatitis with numerous scattered dyskeratotic keratinocytes and confluent partial- to full-thickness epidermal necrosis. Lymphocytes and eosinophils are present within the subepidermal split. There is a mild superficial perivascular lymphocytic infiltrate with scattered eosinophils (H&E, original magnification ×40)

**Fig. 3:**
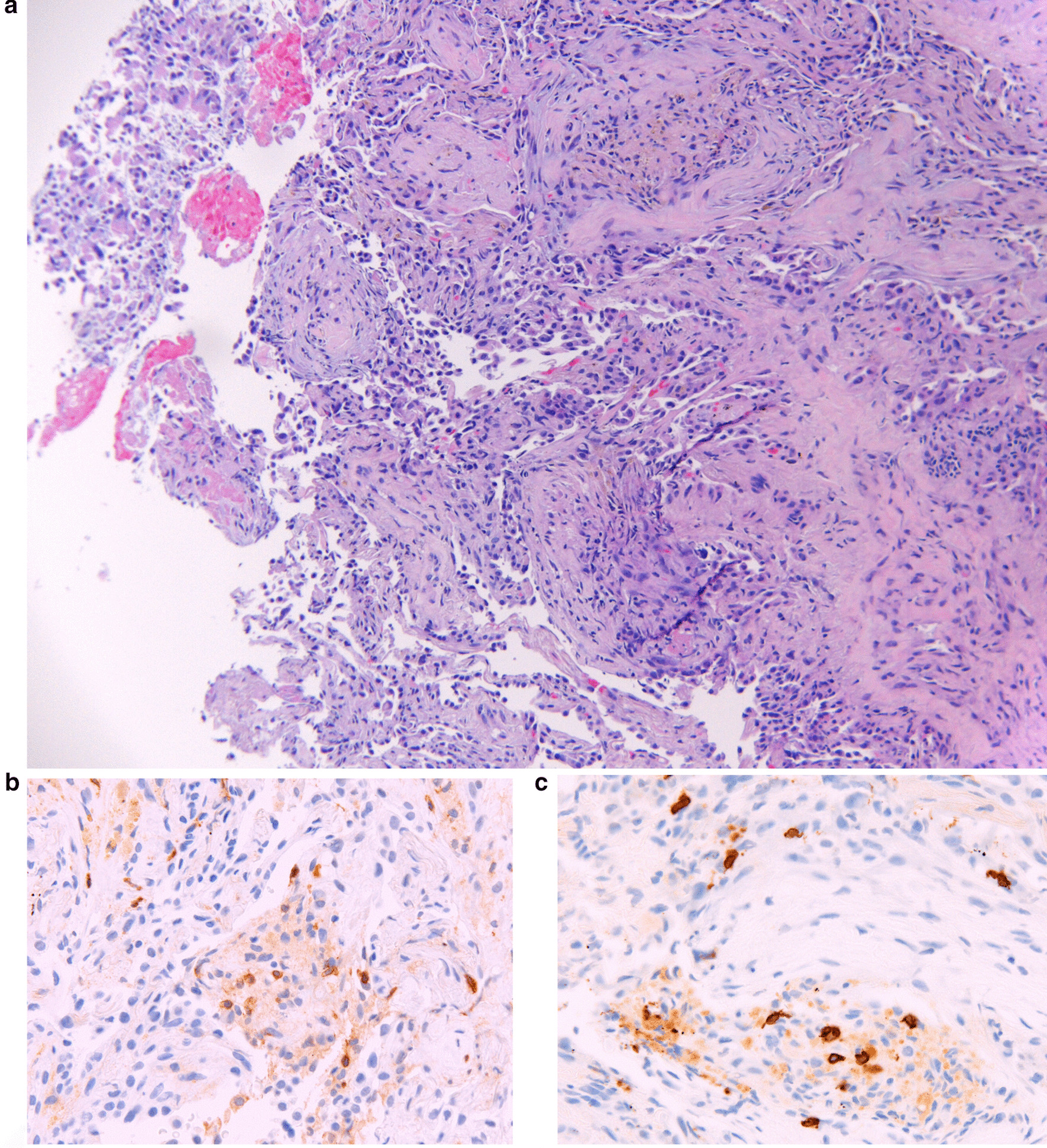
Pathology results of transbronchial biopsy. **a** Higher magnification reveals Masson bodies typical of organizing pneumonia and fibrinous exudate. **b** ×40 magnification of staining for CD4^+^ cells in the tissue and **c** ×40 magnification of staining for CD8^+^ cells in the tissues. Overall, reveals a mixed presence of both CD4^+^ and CD8^+^ in the interstitial infiltrate

However, when the prednisone dose was tapered to 40 mg daily, and over the span of another month, he had a dramatic resurgence of his diarrhea, new painful penile and oral lesions, and a blossoming diffuse rash. He underwent skin biopsy 10 weeks after his last dose of pembrolizumab after another taper to 20 mg daily prednisone, and pathology revealed bullous erythema multiforme (Fig. 2). Attempts to taper prednisone dosage further continued to be challenging, with refractory ICI toxicity, and azathioprine was utilized for additional immunosuppression and steroid-sparing effect. The patient was, however, admitted with a necrotizing right upper lobe pneumonia that persisted despite broad-spectrum antibiotic therapy. Bronchoscopy with transbronchial lung biopsy revealed AFOP (Fig. 3).

Due to persistent symptoms, concern for active infection, and likelihood that these events represented refractory irAEs, azathioprine was stopped and tacrolimus was initiated for broader anti-T-cell inhibitory activity. He was initially on 1 mg twice daily for 1 month and noticed significant improvement in his skin lesions as well as a decrease in his ongoing diarrhea. His respiratory status was stable on room air. His tacrolimus dose was increased to 2 mg every morning and 1 mg every evening and prednisone was weaned to 10 mg daily, with further improvement in his rash to near resolution, and no new skin lesions developed. He continued on a stable dose of tacrolimus for four more months and then it was able to be discontinued with sustained resolution of his irAEs. His malignancy did eventually progress, but only 2 months after discontinuation of the tacrolimus. With almost 1 year of systemic steroid therapy, our patient developed adrenal insufficiency without signs of immune-related hypophysitis, now managed with replacement-dose hydrocortisone.

## Discussion and conclusions

Our case highlights a unique constellation of adverse events, particularly bullous erythema multiforme and AFOP, despite ongoing systemic B-cell-mediated immunosuppression with rituximab and IVIG. A concurrent presentation of two multisystem rare irAEs itself is also notable. The incidence of all immune therapy-related pneumonitis is estimated at < 5% [13], and ICI-related AFOP specifically is even more rarely reported. It has previously been described in only one case report as an irAE from nivolumab therapy [14]. Similarly, while there have been several case reports of bullous pemphigoid and erythema multiforme as a result of anti-PD-1/PD-L1 therapy [15, 16], bullous erythema multiforme is an infrequent dermatologic irAE [17]. This case raises additional questions regarding the susceptibility of individuals to rare multi-organ irAEs. One could consider that the patient’s history of a hematologic malignancy with antibody-associated neuropathy may have played a role. Further studies investigating the development of irAEs in patients with a history of multiple malignancies (specifically prior hematologic malignancies) are warranted.

The activation of anergic T lymphocytes and cross-reactivity between tumor antigens and similar self-antigen are two potential mechanisms for the pathogenesis of irAEs from PD-1 inhibitor therapy [18]. However, the aspect of the immune system responsible for particular organ-specific and multi-organ irAEs has not yet been fully elucidated. Moreover, our patient had no history of T-cell-mediated autoimmune disease but developed seemingly *de novo* multi-organ T-cell-mediated ICI toxicities.

Additionally, our patient’s symptoms were refractory to high-dose corticosteroids, a typically broadly acting and potent T-cell toxin. There was ultimately a response to tacrolimus, a T-cell-specific immunosuppressant, with initial sustained remission of his malignancy. Perhaps the severity and refractory nature of his irAEs was in part due to concurrent B-cell immunosuppression and may reflect consequences of unchecked cell-mediated immunity or even components of the innate immunity by way of T helper cells. Future studies would investigate any potential correlation to decreased B-cell function and the severity and persistence of irAEs.

Current therapies for irAEs rely primarily on high doses and prolonged courses of systemic steroids, which have toxicities of their own including a potential deleterious impact on tumor response [19, 20]. There is still limited evidence to guide the use of steroid-sparing agents. This case offers critical insights into steroid-sparing options for irAE therapy and raises important questions regarding irAE pathophysiology. Our case further emphasizes the need for better characterization of the distinct immunological mechanisms behind different irAEs, the potential role of T-cell-mediated immunity in multi-organ toxicities, the possible benefit of more specific T-cell therapy, the possible role of B-cell dysfunction in augmenting the development of irAEs, and the need for future multi-institutional collaboration efforts to facilitate study of complex steroid-refractory cases in prospective study on a larger scale.

## Data Availability

No additional data set was used for creation of this manuscript. All information was available from standard documentation in the patient’s electronic medical record.

## Abbreviations

irAEs: Immune-related adverse effects
PD-1: Programmed cell death protein 1
PD-L1: Programmed death-ligand 1
MAG: Myelin-associated glycoprotein
IVIG: Intravenous immunoglobulin
RUL: Right upper lobe
AFOP: Acute fibrinous organizing pneumonia
